# A Smartphone-Based Automatic Diagnosis System for Facial Nerve Palsy

**DOI:** 10.3390/s151026756

**Published:** 2015-10-21

**Authors:** Hyun Seok Kim, So Young Kim, Young Ho Kim, Kwang Suk Park

**Affiliations:** 1 Interdisciplinary Program of Bioengineering, Seoul National University, Seoul 03080, Korea; E-Mail: khs0330kr@bmsil.snu.ac.kr; 2 Department of Otorhinolaryngology, Head and Neck Surgery, Seoul National University, Boramae Medical Center, Seoul 07061, Korea; E-Mail: sossi81@hanmail.net; 3 Department of Biomedical Engineering, College of Medicine, Seoul National University, Seoul 03080, Korea

**Keywords:** facial nerve palsy, smartphone, automatic diagnosis, asymmetry, assessment system

## Abstract

Facial nerve palsy induces a weakness or loss of facial expression through damage of the facial nerve. A quantitative and reliable assessment system for facial nerve palsy is required for both patients and clinicians. In this study, we propose a rapid and portable smartphone-based automatic diagnosis system that discriminates facial nerve palsy from normal subjects. Facial landmarks are localized and tracked by an incremental parallel cascade of the linear regression method. An asymmetry index is computed using the displacement ratio between the left and right side of the forehead and mouth regions during three motions: resting, raising eye-brow and smiling. To classify facial nerve palsy, we used Linear Discriminant Analysis (LDA) and Support Vector Machine (SVM), and Leave-one-out Cross Validation (LOOCV) with 36 subjects. The classification accuracy rate was 88.9%.

## 1. Introduction

Facial nerve palsy is a nervous system disorder where there is loss of voluntary muscle movement in a patient’s face caused by nerve damage. A clinical assessment system of facial nerve palsy is an important tool for diagnosing, monitoring and treating facial nerve palsy. The House-Brackmann (H-B) scale is a widely accepted system that grades the facial function from normal (grade 1) to total paralysis (grade 6) [1]. The disadvantage of the H-B scale is its subjective assessment characteristics, which are not reliable and vary among clinicians.

Recently, many studies have proposed methods to quantify facial nerve palsy using image-processing techniques. Current automatic grading systems can be divided into image or video-based. The main advantages of image-based systems are their low computational costs and ease of use. However, such systems have several limitations because images contain less information than video. This drawback introduces errors in calculating the asymmetric index as well as low reproducibility. In response to these disadvantages, most recent studies have proposed video-based systems. Park *et al*. proposed measuring the asymmetric index in the mouth region using video captured with webcams [2]. They used a thresholding approach based on the HSV space and point-tracking algorithms for lip segmentation and tracking, but the algorithms can be easily affected by the recording environment. In addition, the asymmetry index measured only the mouth region. Wang *et al.* proposed an automatic recognition method from six facial actions using active shape models plus Local Binary Patterns (ASMLBP) [3]. They used images, not videos, and only recognized those patterns of facial movements required to evaluate the diagnosis of facial paralysis. McGrenary *et al*. proposed a system that uses an artificial neural network [4]. They trained the network with maximum displacement values and mean intensities calculated through regional analysis. This system is not automatic and its performance can be affected by the conditions of the recording environment, such as lighting conditions. Shu *et al*. demonstrated a system that uses Multiresolution Local Binary Patterns (MLBP) to extract feature and resistor-average distance to measure the asymmetry of facial movements [5]. A total of 197 videos were obtained from a video camera and validated using Support Vector Machine (SVM). The MLBP-based method achieved 94% accuracy for the overall H-B scale. Although their results estimated the facial palsy grade, they had constraints with the recording video environment. Reilly *et al*. used Active Appearance Models (AAMs) for facial feature localization and extracted the distance between the corners of the mouth and mean smile as features [6]. However, they used a synthesized dataset that was not from real-world data and assessed paralysis of smiling function. All these studies are based on separate systems that cannot be carried by clinicians to evaluate patients. Furthermore, previous studies have not focused on the diagnosis of facial palsy nerve patients from normal state and have assumed that measurements are conducted in constrained environment.

In this study, we propose a smartphone-based facial nerve palsy assessment system to diagnose facial nerve palsy quickly and simply in daily life, similar to using videophone functions. The automatic system is designed to helping both patients and clinicians, who only need to perform three motions to diagnose facial nerve palsy and record progression of the disease. To diagnose facial nerve palsy, we use incremental training of discriminative models to detect facial shape points and extract those features that represent face asymmetry using shape points. 

## 2. Experiment

In this section, we describe an incremental parallel cascade of linear regression for face landmark localization and tracking proposed by Asthana *et al.* [7]. Then, we describe the data acquisition and analysis.

### 2.1. Incremental Parallel Cascade of Linear Regression

There have been many successful facial landmark localization algorithms. In this study, we need a robust tracker because patients have asymmetric facial characteristics. When we attempted to localize facial landmarks using standard AAMS, the performance was good for healthy subjects, but poor for patients. For landmark localization, we choose the algorithm referred to as incremental Parallel Cascade of Linear Regression (iPAR-CLR). This algorithm can train data quickly and add new training samples to the regression functions without re-training on previous information. In this study, we use a 2D-shape model described as:

(1) $$s\left(p\right)=sR\left(\bar{s}+\Phi_{s}g\right)+t$$

where s is the vector that represents the location of feature shapes, $\bar{s}$ is the mean location of the shapes, $\Phi_{s}$ denotes the submatrix of the basis of variations, g is the parameters of non-rigid variations of the shape, **R** is rotation, s is scale, and t is translation. The parameters of the model are  p=[s, R,t,g]. The details for iPAR-CLR can be found in [7]. 

**Figure 1:**
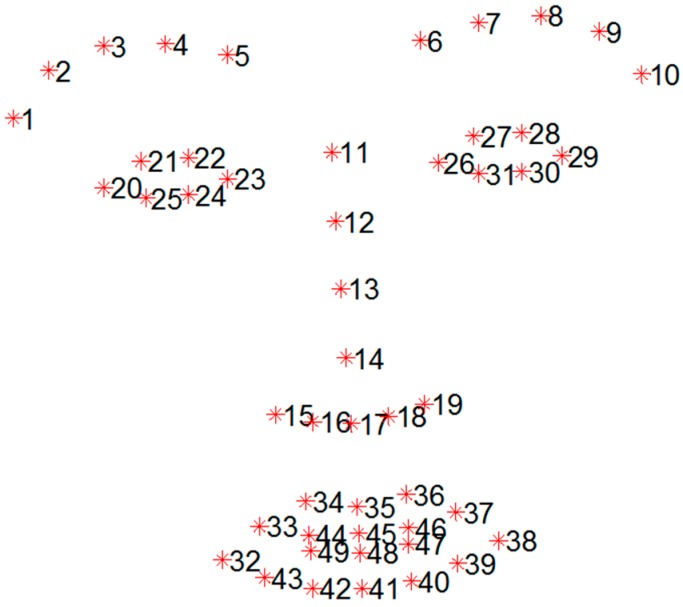
Total of 49 points mark-up used for feature tracking.

**Figure 2:**
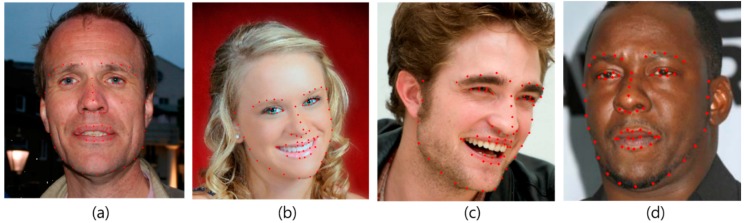
Examples of trained databases. Red points represent 68 annotated landmarks. (**a**) AFW; (**b**) Helen; (**c**) iBUG; (**d**) LFPW.

The facial feature tracker was trained using the iPAR-CLR method with 49 facial landmark points, as shown in Figure 1. For successful facial feature tracking, we used real-world databases not collected in a controlled environment. We trained the facial feature tracker using widely used databases: Labeled Face Parts in the Wild (LFPW) [8], Helen [9], Annotated Faces in the Wild (AFW) [10] and intelligent Behavior Understanding Group (iBUG) [11,12]. LFPW consists of 1423 faces from images downloaded from Google, Flickr, and Yahoo. The Helen database consists of 2330 faces gathered from Flickr. The AFW database consists of 468 faces, and the iBUG database consists of 135 faces. Examples of the trained databases are shown in Figure 2. The databases has 68 annotated landmarks, but we used only 49 points, with the exception of the contour of the face.

### 2.2. Data Acquisition

All participants were asked to perform three facial movements: “rest,” “smile,” and “raise eye brows”. The video was acquired using iPhone 4S and iPhone 6 at 30 frames per second with 1080 × 1920 resolution using a rear-facing camera. The clinicians acquired front view videos of the participants by holding the smartphone in their hands with no constraints and under normal office fluorescent lighting conditions. The video recording duration of each person was 15~20 s.

### 2.3. Feature Extraction

The asymmetric index was calculated using the displacement of shape point sets that correspond to the eye-brows and mouth regions while the participants performed facial movements. To extract the asymmetric index, the forehead and eye regions were used based on the H-B scale and heuristic approach. We applied two approaches local points-based method and axis-based method. Given the 49 landmarks from the facial landmarks localization shown in Figure 1, $P_{i}$ represents the ith landmark and d(x,y) represents the distance between points x and y.

#### 2.3.1. Local Points-Based Feature Extraction

##### Asymmetry Index of Forehead Region

**Figure 3:**
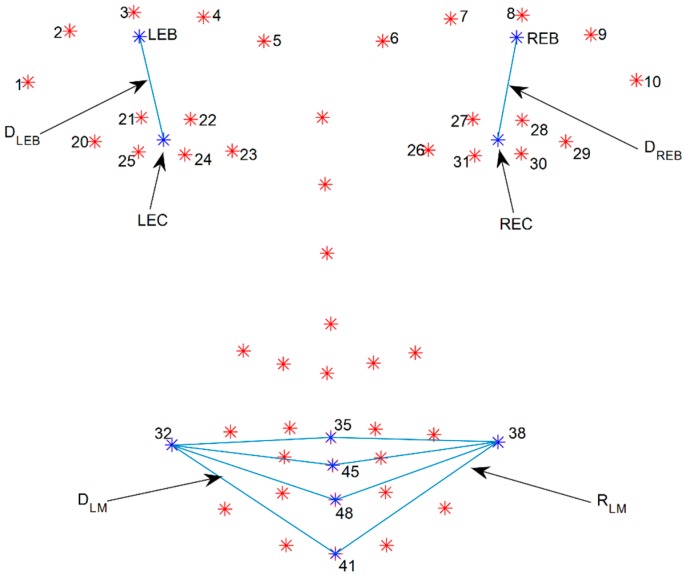
Descriptions of parameters used to calculate asymmetric indices. LEB represents a mean point of left eye brow and REB represents mean point of right eye brow. LEC represents a mean point of left eye and REC represents mean point of right eye. $D_{LEB}$ indicates distance between LEB and LEC. $D_{REB}$ indicates distance between REB and REC. ${\text{P}}_{32}$ represents left mouth corner and ${\text{P}}_{38}$ represents right mouth corner. $D_{LM}$ and $D_{RM}$ indicate mean distance between point of mouth corner (${\text{P}}_{32},{\text{P}}_{38})$ and points of middle of mouth (${\text{P}}_{35},{\text{P}}_{41},{\text{P}}_{45},{\text{P}}_{48}$).

The asymmetry index of the forehead region was calculated using a displacement ratio between the left and right eye brow. We performed the following steps:

(1) Calculate the mean point of the left and right eye-brows (LEB and REB) by averaging five points from each eye-brow from number one to five and six to ten as shown in Figure 3:
  (2) $$\text{LEB}=\frac{1}{5}\overset{5}{\underset{\text{i}=1}{\sum }}{\text{P}}_{\text{i}},\text{ REB}=\;\frac{1}{5}\overset{10}{\underset{\text{i}=6}{\sum }}{\text{P}}_{\text{i}}$$
(2) Calculate the mean point of the eye (LEC and REC) by averaging six points from each eye from the numbers 20 to 25 and 26 to 31 as shown in Figure 3:
  (3) $$\text{LEC}=\frac{1}{6}\overset{25}{\underset{\text{i}=20}{\sum }}{\text{P}}_{\text{i}},\text{ REC}=\;\frac{1}{6}\overset{31}{\underset{\text{i}=26}{\sum }}{\text{P}}_{\text{i}}$$
(3) Calculate distance ($D_{LEB}$ and $D_{REB}$) between the mean point of the eyebrow and that of the eye as shown in Figure 3:
  (4) $$D_{LEB}=\text{d}\left(\text{LEB},\text{LEC}\right),\;D_{REB}=\text{d}\left(\text{REB},\text{REC}\right)$$
(4) Calculate displacement on each side by subtracting the mean distance of the resting state from the maximum distance of the raising eye brow movement:
  (5) $$\begin{array}{c} r_{left\_forehead}=\;\left|\;\max\left(raise_{D_{LEB}}\right)-\text{mean}(\;rest_{D_{LEB}})\;\right|,\\ \;r_{right\_forehead}=\left|\max\left(raise_{D_{REB}}\right)-\text{mean}(rest_{D_{REB}})\;\right| \end{array}$$
(5) Calculate the displacement ratio between the left and right side of the forehead. After comparing the two displacement values, the larger becomes the denominator:
  (6) $$ratio_{forehead}=\{\begin{array}{l} \frac{r_{right\_forehead}}{r_{left\_forehead}},\;\;if\;\;r_{left\_forehead}>\;r_{right\_forehead}\\ \frac{r_{left\_forehead}}{r_{right\_forehead}},\;\;\;\;\;\;\;\;\;\;\;\;\;\;\;\;\;\;\;\;otherwise\;\;\;\;\;\;\;\;\;\;\;\;\;\;\;\;\;\;\;\;\;\;\;\; \end{array}\;\;\;$$

##### Asymmetry Index of Mouth Region

The asymmetric index of the mouth was calculated using the displacement ratio between the left and right mouth corners. We performed the following steps:

(1) Calculate the mean distance ($D_{LM}$ and $D_{RM}$) between the point of the mouth corner and the points of the middle of mouth (${\text{P}}_{35},{\text{P}}_{41},{\text{P}}_{45},{\text{P}}_{48}$) as shown in Figure 3:
  (7) $$\begin{array}{c} D_{LM}=\;\frac{1}{4}\left(\text{d(}P_{32},P_{35}\right)+\text{d(}P_{32},P_{41})+\text{d(}P_{32},P_{45})+\text{d(}P_{32},P_{48}))\\ D_{RM}=\;\frac{1}{4}\left(\text{d(}P_{38},P_{35}\right)+\text{d(}P_{38},P_{41})+\text{d(}P_{38},P_{45})+\text{d(}P_{38},P_{48})) \end{array}$$
(2) Calculate the displacement of each side by subtracting the mean distance of the resting state from the maximum distance of the smile movement:
  (8) $$\begin{array}{c} r_{left\_mouth}=\;\left|\max\;\left(smile_{D_{LM}}\right)-\text{mean}(\;rest_{D_{LM}})\right|,\;\\ r_{right\_mouth}=\left|\max\;\left(smile_{D_{RM}}\right)-\text{mean}(\;rest_{D_{RM}})\right| \end{array}$$
(3) Calculate the displacement ratio between the left and right side of the mouth. After comparing the two displacement values, the larger becomes the denominator.
  (9) ratiomouth={rright_mouthrleft_mouth,  if  rleft_mouth> rright_mouthrleft_mouthrright_mouth,                    otherwise                           

#### 2.3.2. Axis-Based Feature Extraction

**Figure 4:**
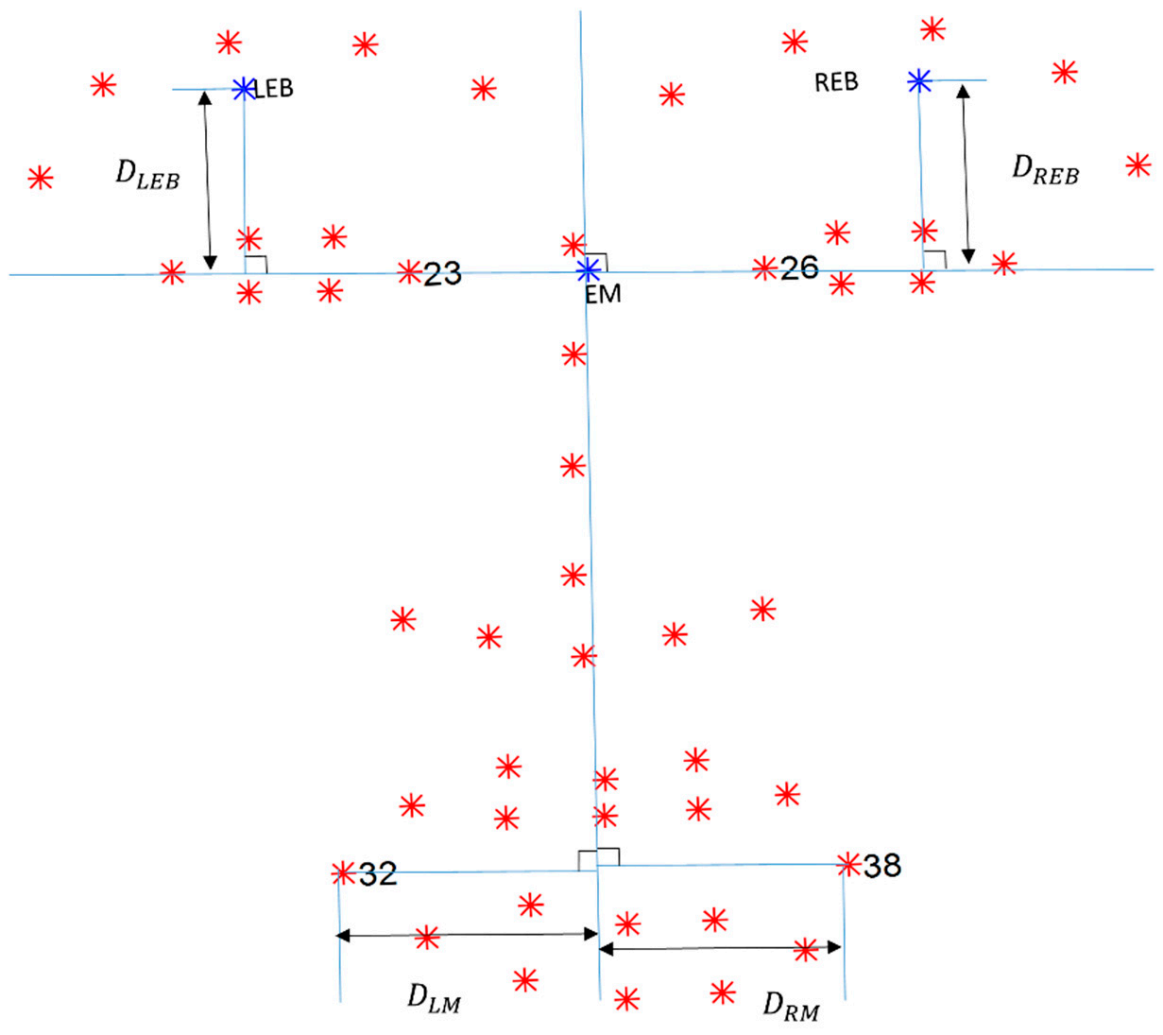
Descriptions of parameters used to calculate asymmetric indices. ${\text{P}}_{23}$ and ${\text{P}}_{26}$ represent left and right medial canthus, respectively. Blue horizontal line is drawn by extending connecting line between ${\text{P}}_{23}$ and ${\text{P}}_{26}$. EM is center point of ${\text{P}}_{23}$ and ${\text{P}}_{26}$. Blue vertical line is drawn perpendicular to horizontal line that passes EM. LEB indicates mean point from left eyebrow. REB indicates mean point from right eyebrow. ${\text{P}}_{32}$ and ${\text{P}}_{38}$ represent left and right mouth corners, respectively. $D_{LEB}$ indicates distance between LEB and point of intersection. $D_{LM}$ indicates distance between point of mouth corner and point of intersection.

The face is divided into the left and right regions based on the shape points of the eyes because they represent the minimum asymmetry among the corresponding regions. First we determine the horizontal line using the points of the eye region, and then calculate the vertical line that is perpendicular to the horizontal line. The horizontal line is the extension of the connected line between the left and right medial canthus (${\text{P}}_{23},{\text{P}}_{26}$) shown in Figure 4. The vertical line is perpendicular to the horizontal line that passes through the eye’s midpoint (EM), which is the mean point of the left and right medial canthus. 

##### Asymmetry Index of Forehead Region

The asymmetry index of the forehead region was calculated using a displacement ratio between the left and right eyebrow. We performed the following steps:

(1) Calculate the mean point of the eyebrows by averaging five points for each eyebrow (from the numbers one to five and six to ten, as shown in Equation (2)).
(2) Find the point of intersection with the mean points of the eyebrows by drawing lines perpendicular to the horizontal line.
(3) Calculate the distance between the mean point of the eyebrow and the point of intersection ($D_{LEB}$ and $D_{REB}$ shown in Figure 4).
(4) Calculate the displacement of each side by subtracting the mean distance of the resting state from the maximum distance of the raising eyebrow movement.
(5) Calculate the displacement ratio between the left and right side of the forehead. After comparing the two displacement values, the larger becomes the denominator.

##### Asymmetry Index of Mouth Region

The asymmetric index of the mouth was calculated using the displacement ratio between the left and right mouth corners. We performed the following steps:

(1) Find the point of intersection with the points of the mouth corners by drawing lines perpendicular to the vertical line.
(2) Calculate the distance between the point of the mouth corner and the point of intersection ($D_{LM}$ and $D_{RM}$ shown in Figure 4).
(3) Calculate the displacement of each side by subtracting the mean distance of the resting state from the maximum distance of the smile movement.
(4) Calculate the displacement ratio between the left and right side of the mouth. After comparing the two displacement values, the larger becomes the denominator.

### 2.4. Subjects

A total of 36 volunteers participated in the study. Of these, 23 subjects suffered from facial nerve palsy and 13 were normal subjects without facial disorders. Before the experiments, each subject was informed of the experiment procedures and the purpose of the study; all consented.

## 3. Results

The images were resized to 540 × 960 to reduce processing time, and asymmetry indices were extracted from the forehead and mouth regions. We compared the performance of combinations of axis and local points-based approaches using Linear Discriminant Analysis (LDA) and SVM with linear kernel as a classification method. Leave-one-out Cross Validation (LOOCV) was used to evaluate the performance of the classification system. Data analysis was performed with MATLAB (Math Works, Inc., Natick, MA, USA).

**Figure 5:**
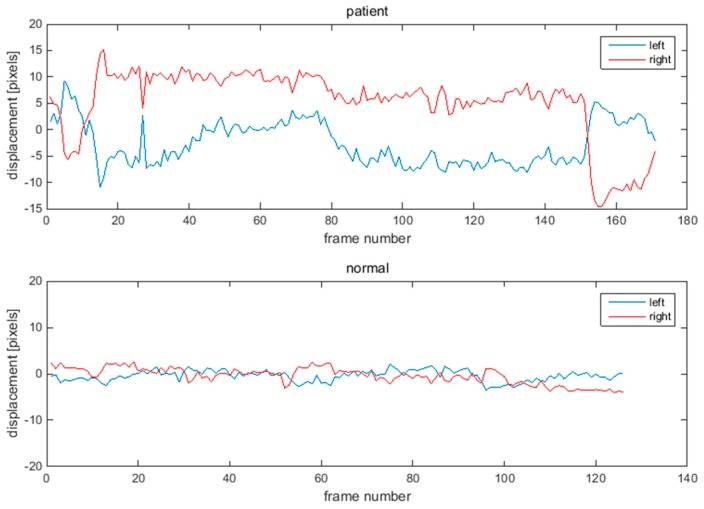
Displacements of eyebrow points during raising eye brow movement. Upper graph shows displacement of a patient. Lower graph shows displacement of a normal subject.

**Figure 6:**
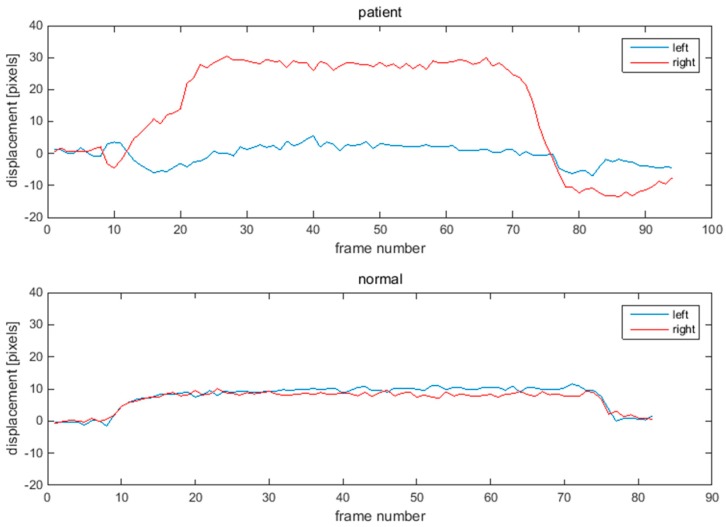
Displacements of mouth corner points during smile movement. Upper graph shows displacement of a patient. Lower graph shows displacement of a normal subject.

Figure 5 and Figure 6 show one example of the displacement of eye-brows and mouths corners for a patient and normal person. The displacement of the normal subject has the symmetry shown in Figure 5 and Figure 6. However, the displacement of the patient with facial nerve palsy has significant asymmetry.

**Figure 7:**
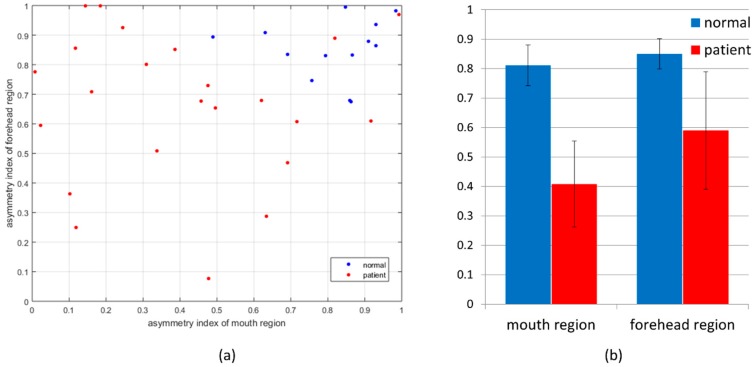
(**a**) 2D plot of asymmetry indices for forehead and mouth region for normal subjects and patients; (**b**) Bar graphs show mean values and standard deviation of asymmetry indices for forehead and mouth regions for normal subjects and patients.

Figure 7 shows that the results of the calculated asymmetry indices with the best combination are the local points-based approach for the forehead and the axis-based approach for the mouth region. The 2D plot of the asymmetry indices of the forehead and mouth regions shows that the mouth region has a more discriminating index. When compared using the mean value and standard deviation of the asymmetry index, the mouth region shows more discrimination than the forehead region. Table 1 represents the classification accuracy, precision rate, and recall rate for LDA and, SVM with linear kernel. The asymmetry index of the forehead region based on local points that of the mouth region based on axis scored the highest classification accuracy at 88.9%. The precision and recall rates were 92.3% and 90.0%, respectively. SVM with RBF and polynomial kernels, not listed in the table, were applied as classifiers, but their results were lower than LDA and SVM with linear kernel.

**Table 1:** Comparison of classification accuracy, precision and recall rate for LDA and SVM with linear kernel on combinations of asymmetry indices.

	LDA			SVM (Linear)		
	Accuracy	Precision	Recall	Accuracy	Precision	Recall
**Forehead_axis + Mouth_axis**	77.8	76.9	66.7	77.8	76.9	66.7
**Forehead_axis + Mouth_region**	66.7	46.2	54.6	63.9	46.2	50.0
**Forehead_region + Mouth_axis**	88.9	92.3	80.0	88.9	92.3	80.0
**Forehead_region + Mouth_region**	75.0	85.7	63.2	77.8	84.6	64.7

## 4. Discussion

### 4.1. Simulation of Asymmetry Index with Various Head Orientations

**Figure 8:**
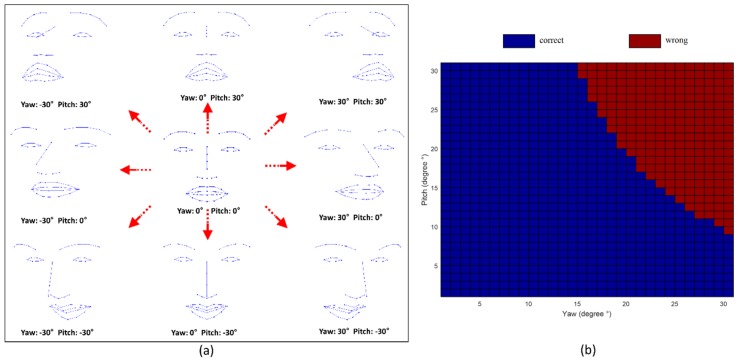
Simulation results of estimating facial nerve palsy among various head pose. (**a**) Examples of projected 2D synthetic face with various head-orientations; (**b**) Simulation results of facial nerve palsy estimation by using best features (forehead_region, mouth_axis) and LDA. The blue color represents the correct estimation, and the red color represents wrong estimation. The results of roll movements are not showed in this figure because there was no change in the asymmetry index within the roll angle from −30° to 30°.

To simulate various head orientations, we used the 3D facial points of a symmetric face consisting of 49 points. The synthetic face was rotated from −30° to 30° at intervals of one degree about the x-, y-, and z- axes, corresponding roll, pitch, and yaw movements. The rotated synthetic face was projected into the x-y plane, which is identical to an image plane, as shown in Figure 8a. We trained the LDA classifier using the best features (forehead_region and mouth_axis) extracted from all participants and tested to synthetic 2D projected face data. According to the simulation results, our method is more sensitive to yaw movement as compared to pitch movement, as shown in Figure 8b. Roll movement did not affect the asymmetry indices.

### 4.2. Measurement Error

During the data analysis, we found that measurement errors lead to wrong classification result. The most critical error occurred when a subject was located on a perfect frontal focus of the camera angle with a rotated head pose. If the left and right mouth corner points moved the same distance while the head rotates at some angle to the camera, the displacement at the image plane of the side rotating toward the camera is increased more than real displacement, whereas the displacement from the side that is rotating away from camera decreases more than the real value. This difference can results in a ratio error calculated from the displacement measurements. If we use the 3D deformable shape model, we can acquire head pose parameters relative to the camera. Therefore, we can calibrate facial landmark positions and reduce the errors in the results caused by head rotation.

The second measurement error is caused by inaccuracy in the facial landmark localization. In general, this error occurs during initialization of the shape points at the start of localization. We can overcome this error by presenting an initial shadow face outline and instructing the subjects to fit their face approximately within the shadow face outline.

### 4.3. Analysis of Eye Region

In this study, we did not analyze closing eye motions for the diagnosis of facial nerve palsy. Calculating asymmetry for the eye region is difficult if displacement of facial points is used. According to the H-B scale, if the eye has asymmetry, the mouth or forehead also has asymmetry. Our goal is to design a system that discriminates facial nerve palsy patients from normal individuals. Within this scope, it is adequate to analyze the forehead and mouth regions only. Shu *et al.* also showed that the forehead and mouth regions have better results than eye closing motions, although the detailed algorithm is different [5].

### 4.4. Combination of Asymmetry Indices

In this study, we used four combinations of asymmetry indices in the forehead and mouth regions using the axis and local points-based approach. The asymmetry index of the local points-based approach in the mouth region exhibited poor accuracy. In the case of normal subjects, the midpoints of the mouth region ($P_{35},P_{41},P_{45},P_{48}$) used as reference points showed few movements during the smile movement. However, in the case of patients, the midpoints were followed the movement of unaffected mouth corner during smile movement, and the distance between the left and right mouth corners had similar values to the reference points. The asymmetry index of the axis-based approach in the forehead region also scored poor accuracy compared with the local points-based approach. We cannot explain the reason for this clearly, but we assume that selecting the eye center as the reference point for the local points-based approach is more suitable than the horizontal line used in the axis-based approach. To calculate each eye center, we used six points, but to obtain the horizontal line, we only used two points; therefore, the eye center might be a more reliable reference point.

### 4.5. Performance Comparison with Conventional Methods

As public databases for facial nerve palsy are not available, we compared the results reported in other articles. Previous studies only showed the methods used without mentioning their performance. Few papers presented the performance of their proposed systems. He *et al*. reported that approximately 94% accuracy was achieved when using MLBP-based method or the optical flow-based method [5]. Reilly *et al*. reported 88% accuracy when using the AAMs-based method [6]. Previous studies reported only accuracy, not the precision or recall rates, which are important for evaluating the performance of the diagnosis system. The accuracy of the proposed method was lower than that of MLBP or optical flow-based method, but similar to that of the AAMs-based method. However, the MLBP-based method used area information by using the front view of the face. Hence, the drawback of this method is that it is not robust to head poses. Our method in this paper was distinct from the AAMs-based method of Reilly *et al* in that asymmetry indices of forehead and mouth region were proposed and real dataset were used.

### 4.6. Limitations of Proposed System

In this study, we only used simple displacement of specific facial points and did not evaluate reproducibility and repeatability which are very important factors for reliable diagnosis system. Reproducibility can be evaluated through an experiment under changing conditions caused by illumination changes, a video recording captured from different smartphones, *etc*. Repeatability can be calculated by repeated measurements of the same subject over a short period. 

### 4.7. Future Works

An automatic asymmetry grading function for the features extracted from the eye closing motion can be added to the proposed system. In addition, the system’s reproducibility and repeatability will be investigated by adding more subjects.

## 5. Conclusions

We propose a smartphone-based system that discriminates between patients with facial nerve palsy and normal subjects using only three facial movements, resting, smiling, and raising eye-brows, without constraints from the recording environment. This system can be helpful for discriminating and monitoring facial nerve palsy patients in their daily life.
